# An Anaphylactic Encounter With Ferric Gluconate Infusion: A Case Report

**DOI:** 10.7759/cureus.63209

**Published:** 2024-06-26

**Authors:** Mohamed A Ebrahim, Eli A Zaher, Tatev Aloyan, Sharathshiva Valaiyaduppu Subas

**Affiliations:** 1 Internal Medicine, Ascension Saint Joseph Hospital - Chicago, Chicago, USA

**Keywords:** allergy and anaphylaxis, iron, colonoscopy, diverticulosis, iron deficiency anemia

## Abstract

Iron deficiency anemia (IDA) is a prevalent condition globally, often necessitating intravenous iron therapy. We present a case of a 71-year-old female with IDA who experienced a severe anaphylactic reaction shortly after commencing a sodium ferric gluconate complex infusion. Prompt cessation of the infusion and administration of epinephrine with steroids led to rapid recovery. This case underscores the importance of recognizing and managing rare yet potentially life-threatening hypersensitivity reactions to intravenous iron formulations, highlighting the need for vigilance among healthcare providers.

## Introduction

Anemia is one of the most common medical conditions worldwide, affecting about a quarter of the world's population. About half of anemia cases are attributed to iron deficiency. Commonly, the source of iron deficiency anemia (IDA) is blood loss, either as gastrointestinal bleeding in adult males or menstruation in premenopausal females. In those with gastrointestinal bleeding, the prevalence of IDA is over 60%. Although oral iron supplementation is generally adequate, it may be complicated by malabsorption and poor tolerance from escalating required doses for appropriate correction [1]. In such cases, intravenous (IV) iron is the treatment of choice [2]. Such formulations carry a small risk of hypersensitivity reactions, possibly from the dextrans acting as plasma expanders. However, anaphylaxis is extremely rare and is estimated at 1 per 250,000 administrations [3]. 

We report a case of an elderly lady with IDA who developed an anaphylactic reaction shortly after initiation of an intravenous iron infusion.

## Case presentation

A 71-year-old female presented to the Emergency Department with complaints of exertional shortness of breath, fatigue, and melena for the preceding month. Her medical history is significant for gastric bypass surgery performed five years prior and generalized anxiety disorder. She denied having had similar symptoms in the past. No abdominal pain, vomiting, diarrhea, or weight loss was reported. She likewise denied being on any anticoagulation medications, iron pills, or nonsteroidal anti-inflammatory drugs (NSAIDs). She never had a screening colonoscopy. 

Upon admission, her vital signs were within normal limits with adequate oxygen saturation on room air. Physical examination, including rectal inspection, was unremarkable. Laboratory work-up demonstrated acute microcytic anemia. Hemoglobin was 6.7 g/dL (reference range 12.0-15.3) with a mean corpuscular volume (MCV) of 71.0 fL (reference range 80.0-100.0). Red blood cell distribution width (RDW) was elevated to 18.1% (reference range 11.9-15.9), and ferritin was 5 ng/mL (reference range 11-307), pointing towards IDA secondary to blood loss. Her other blood work-ups, including vitamin B12 and folate levels, were normal.

**Figure 1:**
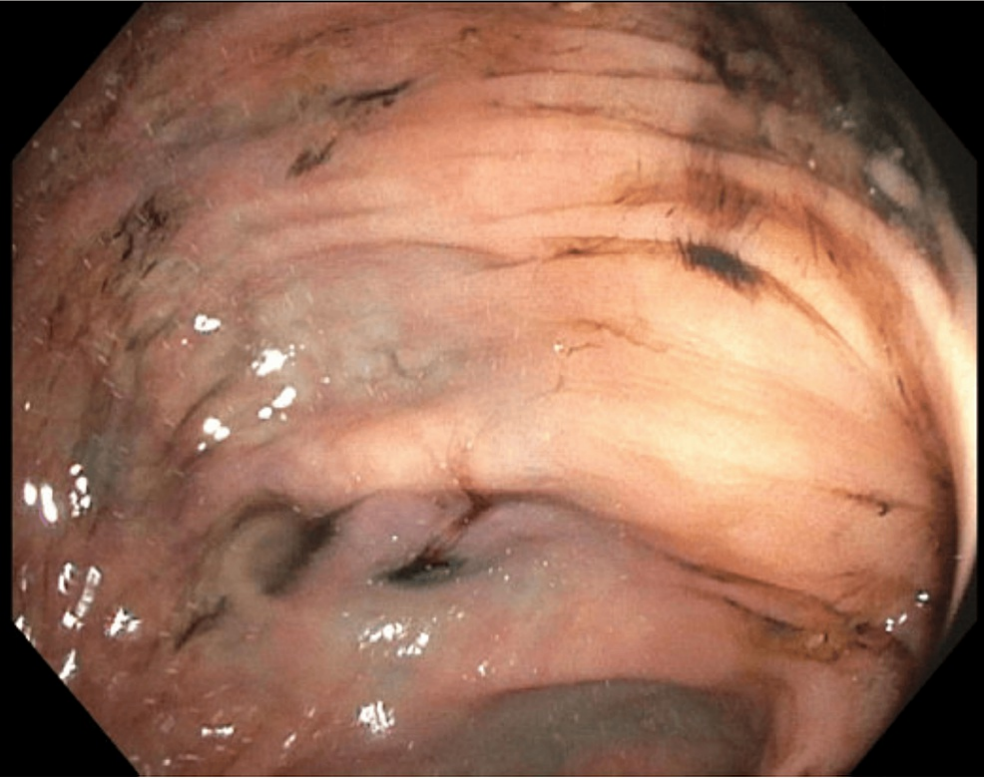
Extensive diverticulosis as seen on colonoscopy

Our patient thus received a unit of blood transfusion with an appropriate hemoglobin elevation of 7.8 g/dL. She underwent a colonoscopy the following day, which revealed significant diverticulosis throughout the colon without an active focus of bleeding (Figure 1).

Simultaneous upper endoscopy was unremarkable. Given a remote history of constipation with oral iron supplementation, she refused oral therapy and opted for IV iron. Shortly after the initiation of the iron infusion, she began feeling chest tightness, back pain, and diffuse itchiness. Blood pressure at the time was 86/54 mmHg with tachycardia to 105 bpm. Physical examination was negative for any skin findings, stridor, or wheezing. The iron infusion was stopped. She received 50 mg of Benadryl, 20 mg of famotidine, 80 mg of methylprednisolone, 0.3 mg of epinephrine, and a liter bolus of lactated Ringer’s solution, with rapid recovery. Following an additional day of observation, she was discharged home in good health on oral iron and laxatives.

## Discussion

Anemia, marked by a reduction of red blood cells or hemoglobin in the bloodstream, is a widespread condition affecting diverse populations. Among its various forms, IDA emerges as the primary culprit globally. This deficiency not only impacts individual health but also poses significant challenges to healthcare systems worldwide [3]. Oral iron supplements can often be ineffective due to malabsorption or non-compliance, making parenteral therapy necessary [4]. In the United States, several types of IV iron therapy are available: iron dextran, sodium ferric gluconate complex (SFGC), iron sucrose, ferumoxytol, ferric carboxymaltose, and ferric derisomaltose [1]. While all effectively increase hemoglobin levels, their safety profiles vary significantly. Iron dextran has been associated with over 30 deaths in the past two decades due to anaphylactic reactions, likely from the dextran component [5]. Conversely, SFGC and iron sucrose have not been linked to any fatalities. Iron sucrose is well-studied and commonly used in Europe and the Middle East [5,6].

Although SFGC has not caused any deaths, adverse reactions such as allergic skin reactions, hypotension, vomiting, diarrhea, headache, and myalgias occur at a rate of about 3.3 per million doses annually, compared to 8.7 per million for iron dextran [5]. Most reactions are mild and self-limiting, but both SFGC and iron dextran can cause life-threatening anaphylactoid reactions. In hemodialysis patients, iron dextran showed a 0.6% incidence of severe reactions, whereas SFGC showed a significantly lower rate of 0.04%, making it safer for treating IDA in chronic renal failure [2,6]. Symptoms of anaphylaxis, such as pruritus, angioedema, laryngeal edema, hypotension, and abdominal cramping, typically occur within minutes of exposure and require immediate cessation of the medication [7].

The mechanisms by which iron infusions cause adverse reactions may vary depending on the iron formulation and the patient's underlying conditions. These reactions often present similarly but can be triggered by different mechanisms, such as IgE-mediated responses to dextran components or complement activation-related pseudoallergy (CARPA) [8]. Current data do not support IgE-mediated hypersensitivity as a common cause of reactions to modern IV iron formulations [9]. CARPA, involving nanoparticle-containing infusions, is likely the most common mechanism, leading to mast cell and basophil activation and releasing substances that cause symptoms like bronchospasm, edema, and shock [8]. The following steps are warranted as a part of the management of a severe anaphylactoid reaction from IV iron causing a life-threatening hypersensitivity reaction: administer an intramuscular injection of adrenaline into the anterolateral thigh at a dose of 0.3 to 0.5 mg. Continuous monitoring of ECG and blood pressure is crucial to detect potential arrhythmias or a hypertensive reaction to adrenaline. Provide high-flow oxygen (>10 L/min) via a face mask initially, along with nebulized β2-adrenergic agonist and/or ipratropium to alleviate wheezing if present. Administer rapid volume expansion with 1-2 L of 0.9% saline or similar isotonic fluid. If not previously administered, give IV corticosteroids as part of managing a moderate hypersensitivity reaction. Following successful initial interventions, closely monitor the patient for at least four hours after symptom resolution, with potential observation extending up to 24 hours [10].

IV iron therapies are linked to an estimated incidence of severe adverse events of less than 1 in 250,000 doses and rarely cause anaphylaxis. Population-based studies in the United States have indicated that the risk of anaphylaxis ranges from 4.0 to 6.8 per 10,000 first administrations of IV iron dextran and from 2.0 to 2.4 per 10,000 first administrations of non-dextran IV iron. However, population studies in Europe are not available. Between January 1, 2014, and December 31, 2019, the US Food and Drug Administration Adverse Event Reporting System database, which is based on post-authorization spontaneous reports, recorded 57 hypersensitivity reactions and 22 anaphylactic reactions with four deaths associated with IV iron dextrans; 212 hypersensitivity reactions and 43 anaphylactic reactions with seven deaths linked to IV iron sucrose; 196 hypersensitivity reactions and 67 anaphylactic reactions with 23 deaths related to IV ferumoxytol; and 401 hypersensitivity reactions and 51 anaphylactic reactions with one death associated with IV ferric carboxymaltose [11]. 

## Conclusions

Hypersensitivity reactions to IV iron infusions are infrequent yet potentially life-threatening occurrences. Our case illustrates that a severe response to SFGC therapy is possible, emphasizing the importance of promptly identifying and managing it appropriately to ensure favorable results. There is a scarcity of evidence regarding the management of hypersensitivity reactions to iron infusions. Given the rarity of these reactions, conducting formal clinical trials to assess optimal therapeutic approaches is not practical. However, areas necessitating further investigation include understanding the underlying causes of hypersensitivity reactions, defining risk factors in individual patients and various diseases, and evaluating the effectiveness of any pre-medication. Managing these reactions requires prompt recognition and assessment of severity, along with vigilant monitoring and immediate intervention. All personnel involved in administering iron infusions should be aware of alarming signs to ensure prompt management.
